# Patellar Cartilage Bossing Causing Patellofemoral Pain After Cartilage Repair With Hyalofast® Scaffold and Bone Marrow Aspirate Concentrate (BMAC)

**DOI:** 10.7759/cureus.43967

**Published:** 2023-08-23

**Authors:** Wei Lun Cheong, Hamid Rahmatullah Bin Abd Razak

**Affiliations:** 1 Orthopaedic Surgery, Sengkang General Hospital, Singapore, SGP; 2 Musculoskeletal Sciences, Duke-Nus Medical School, Singapore, SGP

**Keywords:** cartilage bossing, arthroscopy, bone marrow aspirate concentrate, chondral lesions of knee, hyalofast

## Abstract

Chondral lesions of the knee are common orthopedic issues encountered in daily practice. Hyalofast^®^ (Anika Therapeutics, Bedford, Massachusetts) grafting with bone marrow aspirate concentrate (BMAC) has been shown, in numerous studies, to be clinically efficacious in the surgical treatment of these lesions. We describe a case of chondral lesions in the knee initially successfully treated with arthroscopic chondroplasty and Hyalofast^®^ grafting with BMAC, but who subsequently developed patellar cartilage bossing, causing anterior patellofemoral joint pain eight months after surgery. A 48-year-old gentleman underwent arthroscopic chondroplasty and mini-open cartilage repair with Hyalofast^®^ and BMAC after presenting with right knee pain and swelling affecting his ability to participate in sports. His initial recovery was unremarkable, but he subsequently developed anterior knee pain exacerbated by stair climbing and jogging eight months after the initial surgery. Magnetic resonance imaging revealed bossing of the Hyalofast^®^ graft over the central patella, and intraoperative testing confirmed the impingement. The patient’s symptoms resolved immediately following arthroscopic debridement. While Hyalofast^®^ grafting and BMAC remain viable options in the treatment of chondral lesions in the knee, the inability to predict the amount of cartilage repair and recovery may result in pain and impingement symptoms, and surgeons should be aware of such possible complications.

## Introduction

Articular cartilage injuries are common complaints encountered in the orthopedic surgery clinic. Full thickness chondral lesions are found in 5-10% of knees undergoing arthroscopy surgery [1]. Due to its avascular and aneural properties, articular cartilage has poor potential for healing and recovery [2]. Many different surgical treatment strategies have been described, including chondroplasty, drilling and microfracture, mosaicplasty, and autologous chondrocyte implantation [3-5]. Hyalofast^®^ (Anika Therapeutics Inc, Bedford, MA) is a non-woven biodegradable hyaluronic acid (HA)-based scaffold for hyaline-like cartilage regeneration, entrapping mesenchymal stem cells (MSCs) to treat both chondral and osteochondral lesions in the knee and ankle. As the Hyalofast^®^ scaffold degrades, it exudes more HA into the lesion, resulting in an embryonic-like microenvironment that further promotes cartilage growth [6].

In this case report, we present a rare case of a patient who was initially successfully treated with Hyalofast^®^ and BMAC but subsequently developed patellofemoral anterior knee pain as a result of cartilage bossing. The objective of this case report is to highlight an uncommon complication that may arise due to an inability to predict the amount of cartilage repair and recovery following Hyalofast^®^ grafting with BMAC.

## Case presentation

A 48-year-old gentleman of Indian ethnicity presented to the clinic with a two-month history of right knee pain. He was an avid amateur badminton player and developed lateral knee pain acutely during a game. He subsequently developed swelling over the anterolateral aspect of the knee. The pain became recurrent and was exacerbated by his badminton games. He denied any trauma, instability, or locking symptoms. He reported a pain visual analog scale (VAS) score of six.

Clinical examination revealed localized swelling over the anterolateral joint line. There was no joint line tenderness. Knee cruciate and collateral ligament examination did not reveal any instability. The knee range of motion was from 0 to 90 degrees. Magnetic resonance imaging (MRI) examination of the right knee was performed. It revealed a 7 mm loose body inferolateral to the patella against the lateral femoral condyle, as well as chondral erosion in the apex of the patella extending to the medial facet. The patient was offered both conservative and surgical options. Conservative management would involve physical therapy and pharmacological treatment, whereas surgical management would be an arthroscopic removal of loose body and mini-open cartilage repair with Hyalofast^®^ and BMAC. Known effective conservative management includes weight loss, nutrition, and physical activity, and tends to be more successful in younger patients with mild to moderate cartilage defects [7]. Dekker et al. recommended surgical management, either via osteochondral autograft transfer (OAT), autologous chondrocyte implantation (ACI), matrix-assisted ACI (MACI), or newer biologic scaffolds of allograft cartilage in younger or athletic patients [4]. Migliorini et al., in their systemic review, noted that the clinical outcomes were mostly related to the patient’s performance status prior to surgery [8]. As the patient was very keen to return to badminton and was very active pre-injury, he opted for surgical management. Chondroplasty was performed arthroscopically. BMAC was harvested from the patient’s distal femur. The Hyalofast^®^ graft was prepared and applied to all the chondral defects. BMAC was then applied on top of the grafts. The right knee was kept in a brace and allowed range of motion from 0 to 30 degrees strictly. He was not allowed to weight bear on the right knee for six weeks and was reviewed regularly in the clinic. 

**Figure 1:**
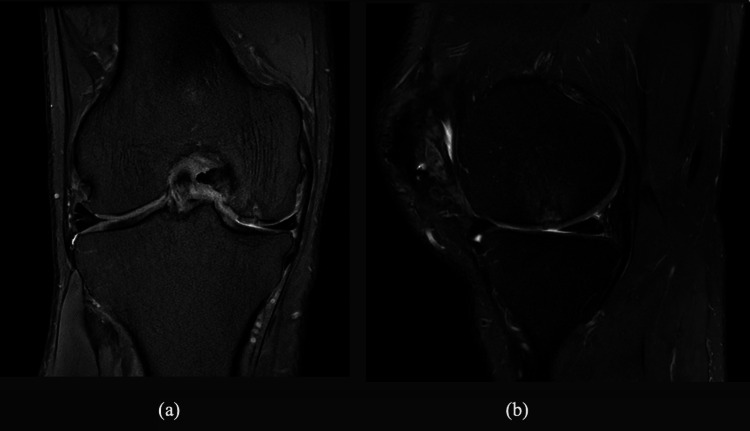
(a-b) MRI T2-weighted images of the coronal and sagittal cuts revealing the Hyalofast® patch located at the posterior weight-bearing surface of the medial femoral condyle The Hyalofast^®^ patch shows adherence to the bony surface and is of similar thickness to the surrounding native articular cartilage.

Six months after the surgery, the patient returned to light jogging and was able to jog about two kilometers without pain. However, about eight months postoperatively, he started to develop anterior knee pain over the patellofemoral joint. This pain was exacerbated by stair climbing and jogging. Clinical examination was otherwise unremarkable. A bedside sonography examination was performed, which revealed possible scar tissue or cartilage flap in the lateral suprapatellar region. In view of the above finding, an MRI examination with cartilage mapping was performed. The Hyalofast^®^/BMAC patches at the central patella, femoral trochlear, and medial femoral condyle remained adherent to the bone with minimal gap between the deep patches and native articular cartilage, as depicted in Figures 1a, 1b.

**Figure 2:**
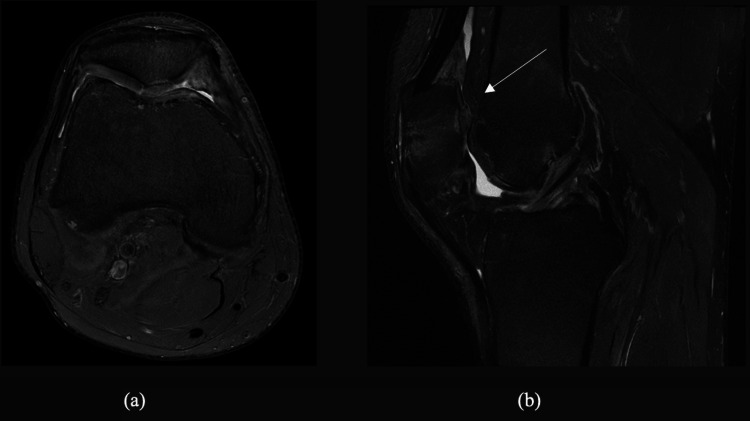
(a-b) MRI T2-weighted images of the axial and sagittal cuts revealing adherence of the Hyalofast® patch to the bone surface The white arrow indicates the area of the ‘bossing’ of the Hyalofast^®^ patch, showing increased thickness by 1.7 mm.

The central patellar Hyalofast^®^ patch, however, showed mild surface bossing with increased thickness compared to the surrounding native articular cartilage in Figure 2a. The cartilage surface showed areas of irregularity in Figure 2b.

**Figure 3:**
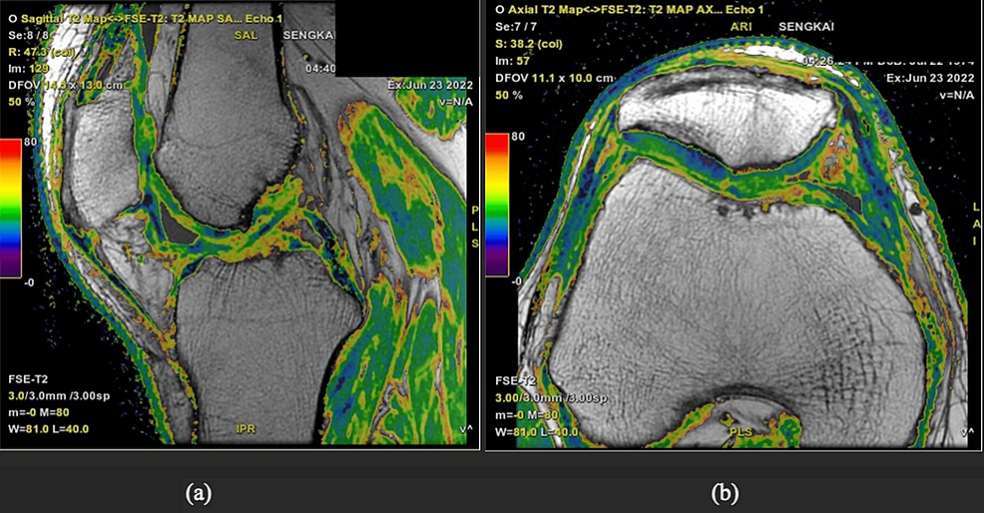
(a-b) Cartilage mapping sequence on MRI showing the patch having a heterogenous signal, slightly lower in signal intensity towards the base The deep layer T2RV is generally similar to the adjacent native articular cartilage, whilst the surface layer shows foci of mid to high T2RV. T2RV: T2 relaxation values

The cartilage mapping sequence revealed a heterogenous signal throughout the entire patch, with the deeper layer showing a similar signal to the adjacent native articular cartilage, while the surface layer showed higher signal intensity. This was seen in the sagittal cut in Figure 3a and the axial cut in Figure 3b. 

**Figure 4:**
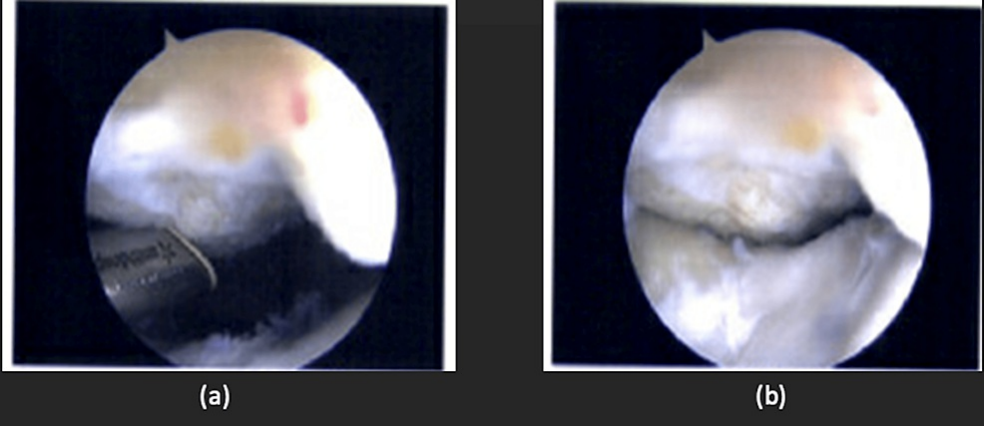
(a-b) Arthroscopic images showing cartilage overgrowth over the previous patella cartilage repair site, with impingement on the femoral trochlea.

The patient was then offered surgical arthroscopic debridement and chondroplasty in view of the clinical and radiological findings corroborating with his clinical symptoms. Intraoperative findings revealed cartilage overgrowth over the previous patella cartilage repair site, with impingement on the femoral trochlea, as seen in Figures 4a, 4b. The previous trochlea cartilage repair site had good cartilage coverage, with slight recessing from the surrounding native cartilage. Previously noted chondral lesions in the medial and lateral tibiofemoral compartments did not show any deterioration.

**Figure 5:**
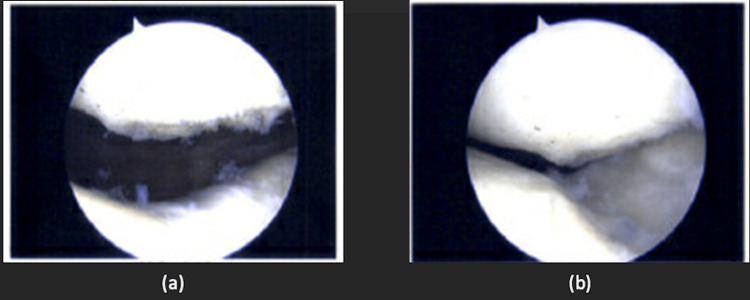
(a-b) Arthroscopic images showing post-debridement of the cartilage overgrowth, with the removal of impingement upon dynamic testing

The overgrown cartilage was trimmed with both the arthroscopic shaver and radiofrequency wand until the level of surrounding native cartilage, depicted in Figures 5a, 5b. Dynamic testing intraoperatively did not reveal any impingement following chondroplasty. He was allowed the full range of motion and weight-bearing as tolerated postoperatively.

The patient was reviewed two weeks after surgery. He was well with minimal postoperative pain and was able to range his knee pain-free from 0 to 90 degrees. He reported a pain VAS score of one. Unfortunately, the patient was lost to follow-up after two weeks and did not return for his scheduled appointments.

## Discussion

Hyalofast^®^ grafting combines a hyaluronic-acid scaffold with MSCs derived from BMAC. Buda et al. showed improved International Knee Documentation Committee (IKDC) and Knee injury and Osteoarthritis Outcome Score (KOOS) scores in 30 patients with osteochondral lesions of the knee treated with Hyalofast^®^ at three years of follow-up, showing the viability of Hyalofast^®^ as a surgical option for these knee conditions [9]. In patients with Grade IV knee articular cartilage ulcers, a Hyalofast^®^ graft with microfracture has been shown to improve KOOS scores in symptoms, pain, and daily living categories [10]. When compared with microfracture alone, patients undergoing Hyalofast^®^ graft surgery with BMAC showed better clinical outcomes and more durable cartilage repair at five years, and the improved outcome was independent of age or lesion size [11]. When specifically looking at full-thickness chondral defects of the knee in patients above 45 years old, all scores significantly improved at the four-year follow-up. MRI showed complete cartilage filling in 80% of the patients. In particular, this study showed that the efficacy of Hyalofast^®^ graft treatment was not affected by age but was mainly affected by lesion size and number [12]. In terms of long-term outcome, Gobbi et al. followed up on 23 patients with full-thickness chondral injuries treated with a Hyalofast^®^ graft with BMAC for a mean of eight years and showed good to excellent clinical outcomes in the treatment of small to large lesions. No associations, however, were identified between body mass index or lesion size and outcome scores [13]. Good outcomes with the use of the Hyalofast^®^ graft have also been reported in the treatment of chondral injuries of other joints [14,15].

In our presented case, our patient showed good initial recovery with his arthroscopic chondroplasty, Hyalofast^®^ grafting, and BMAC. However, he developed patellofemoral pain subsequently. Sonographic and MRI examination revealed good adherence and healing of the central patella, femoral trochlear, and medial femoral condyle where Hyalofast^®^ grafting with BMAC was performed. However, surface bossing of the Hyalofast^®^ patch over the central patella was noted. Intraoperative testing revealed it as being a source of impingement, and the patient did experience relief of symptoms following surgical debridement. Interestingly, histological assessment of the debrided tissue showed a mixture of fibrous and hyaline-like cartilage, likely representative of ongoing re-modeling of the cartilage repair site.

## Conclusions

In conclusion, the use of Hyalofast^®^ grafting with BMAC has shown promising results in the treatment of chondral lesions of the knee. We describe a rare case of overgrowth of repair tissue after arthroscopic chondroplasty and Hyalofast^®^ grafting with BMAC, which led to anterior patellofemoral knee pain and impingement. The inability to predict the amount of cartilage repair and recovery following Hyalofast^®^ grafting with BMAC may result in pain and impingement symptoms, which this case report aims to highlight. Surgeons performing such procedures should be aware of these complications.
